# Safety measures for COVID-19: a review of surgical preparedness at four major medical centres in Saudi Arabia

**DOI:** 10.1186/s13037-020-00259-1

**Published:** 2020-09-05

**Authors:** Mohammad A. Alsofyani, Haifaa M. Malaekah, Ahmed Bashawyah, Mohammed Bawazeer, Khalid Akkour, Sultan Alsalmi, Abdu Alkhairy, Nayef Bin Dajim, Salahaddeen Khalifah, Ibrahim A. Almalki, Farid Kassab, Mohammad Barnawi, Mosfer Almalki, Mohammed Alharthi, Majed Alharthi, Abdulaziz Almalki, Abdullah H. Almalki, Anouar Bourghli, Ibrahim Obeid

**Affiliations:** 1 Orthopedic Department, College of Medicine and University Hospital, University of Hail, Hail, Kingdom of Saudi Arabia; 2 General Surgery Department, King Abdullah Bin Abdulaziz University Hospital, Princess Nourah Bint Abdulrahman University, Riyadh, Kingdom of Saudi Arabia; 3 Anesthesiology Department, College of Medicine and King Abdulaziz University Hospital, Jeddah, Kingdom of Saudi Arabia; 4 Critical Care Medicine, King Faisal Specialist Hospital and Research Center, Riyadh, Kingdom of Saudi Arabia; 5 Obstetrics and Gynecology Department, King Saud University, Riyadh, Kingdom of Saudi Arabia; 6 Department of Neurosurgery, Imam Abdulrahman Bin Faisal University, Dammam City, Kingdom of Saudi Arabia; 7 Department of Neurosurgery, King Faisal Medical City, Abha, Kingdom of Saudi Arabia; 8 Neuroscience Center, King Abdullah Medical City, Makkah, Kingdom of Saudi Arabia; 9 Infection Control Department, Mental Health Hospital, Taif, Kingdom of Saudi Arabia; 10 Musculoskeletal Center of Excellence, International Medical Center, Jeddah, Kingdom of Saudi Arabia; 11 Anesthesia Department, College of Medicine and University Hospital, Albaha University, Albaha, Kingdom of Saudi Arabia; 12 Hematology Oncology Department, King Faisal Specialist Hospital and Research Center, Jeddah, Kingdom of Saudi Arabia; 13 Biochemistry and Molecular Medicine Department, College of Medicine, Taibah University, Madinah, Kingdom of Saudi Arabia; 14 General Surgery Department, Security Forces Hospital, Makkah, Kingdom of Saudi Arabia; 15 Urology Department, King Faisal Hospital, Taif, Kingdom of Saudi Arabia; 16 Physical Medicine and Rehabilitation, Armed Forces Rehabilitation Center, Taif, Kingdom of Saudi Arabia; 17 Orthopedic and Spinal Surgery Department, Kingdom Hospital, Riyadh, Kingdom of Saudi Arabia; 18 Spine Surgery Department, Specialist Terrefort Clinic, Bruges, France

**Keywords:** COVID-19, Anaesthesia, Surgery, Guideline, Simulation

## Abstract

In view of the worldwide coronavirus disease 2019 (COVID-19) pandemic, hospitals need contingency planning. This planning should include preparation for an unexpected patient surge. This measure is evolving concomitantly with the implementation of the needed infection control rules. Here, we present our experience in contingency planning at four large tertiary hospitals in Saudi Arabia during this global pandemic, with a focus on dealing with COVID-19 patients who need to undergo surgery. The planning covers response measures required in the operating room and supporting units, including the administrative department, intensive care unit, and different sections of the surgical department. Furthermore, it covers the role of education and simulation in preparing health care providers and ensuring smooth workflow between all sections. We additionally discuss the guidelines and policies implemented in different surgical specialties. These measures are necessary to prevent the transmission of COVID-19 within healthcare facilities. Throughout the COVID-19 pandemic, the healthcare system should develop a comprehensive pandemic plan and set guidelines addressing the management of urgent and malignant cases. The guidelines should be in concordance with internal guidelines.

## Background

Coronavirus disease 2019 (COVID-19) was first recorded as a case of pneumonia caused by an unknown pathogen in Wuhan, the capital city of the Hubei Province in China, in December 2019 [1]. Coronaviruses are a group of enveloped, positive-single-stranded RNA viruses [2]. The coronavirus that causes COVID-19 is known as severe acute respiratory syndrome coronavirus 2 (SARS-CoV-2). SARS-CoV-2 is mainly transmitted through respiratory droplets and person-to-person contact; however, past experience with SARS-CoV in 2003 suggests the possibility of airborne spread in a relatively closed area—i.e., when individuals are exposed to high concentrations of aerosols for a long time, most commonly following tracheal intubation [3]. COVID-19 symptoms are similar to those of other upper respiratory diseases: fever, cough, dyspnoea, and fatigue [4]. However, the symptoms can be nonspecific, especially in elderly and immunocompromised patients [5]. On 30 January, 2020, the World Health Organization (WHO) pronounced COVID-19 a public health emergency of international concern [6], with a high economic global impact. On 11 March, 2020, the WHO declared COVID-19 to be a pandemic [7]. With the increasing number of diagnosed cases, a COVID-19 pandemic is becoming a reality. Updated knowledge on the biology and transmission of this virus is limited [8, 9]; therefore, estimating the fatality rate this early in the outbreak is difficult. Early fatality estimates often exclude mild cases and fail to consider that some infected patients might die thereafter.

The Ministry of Health (MOH) in the Kingdom of Saudi Arabia (KSA) officially announced the detection of the first COVID-19 case on 2 March, 2020. By 9 March, 2020, the number of cases had risen to 20, and the government decided to cancel all social events and to close educational institutions and governmental agencies to control infection spread. On 21 March, 2020, the KSA announced the suspension of all flights, buses, and trains to and from the KSA and between its major cities. Moreover, the government isolated all currencies imported from outside the Kingdom. Additionally, it imposed a public curfew starting from 19:00 until 06:00 daily, which was further extended from 15:00 to 06:00 in all cities.

Operating rooms (ORs) are potential risk areas for the transmission of airborne infections due to the presence of a multidisciplinary team and the requirement for high-transmission risk activities, such as airway assessment. Additional challenges presented by the COVID-19 outbreak include the global demand for resources, staff burnout, increased transmission risk, and increased load on our health care systems. Anaesthesiologists are in close contact with the patient’s airway and consequently face a high risk of airborne transmission. Using a procedure-specific protocol for surgical anaesthesia for COVID-19 patients will help reduce potential cross-infections in the hospital. This review aims to share our experience of dealing with COVID-19 patients requiring surgery in the tertiary hospitals of the KSA. The measures taken for preventing cross-infections include the designation and preparation of an isolation surgical theatre, remodelling of surgical plans and processes, staff organisation, and adjustment of surgical and anaesthetic guidelines.

### Hospital facilities related to administration and human resource management

The KSA government decided to establish a national command and control centre with the following aims: 1) monitor and follow up cases, 2) plan preventive measures based on national and worldwide daily updates of COVID-19, 3) limit viral spread within and beyond the hospitals, 4) optimise the hospital workforce in anticipation of the COVID-19 outbreak, and 5) distribute limited resources in a rational, ethical, and organised way.

The first step was assigning a certain location for COVID-19 patients to prevent cross-contamination. If the hospital was composed of well-separated buildings, certain buildings were allocated for COVID-19 patients. If the hospital was composed of a single building, certain floors were entirely allocated for COVID-19 patients.

At the administrative level, the command centre was established to exclusively deal with COVID-19 patients. The command centre chief had direct access to the chief executive officer. One medical officer responsible for all patients on the medical floors, one dealing with intensive care unit (ICU) patients, and one dealing with nursing issues reported to the chief of the command centre.

**Fig. 1:**
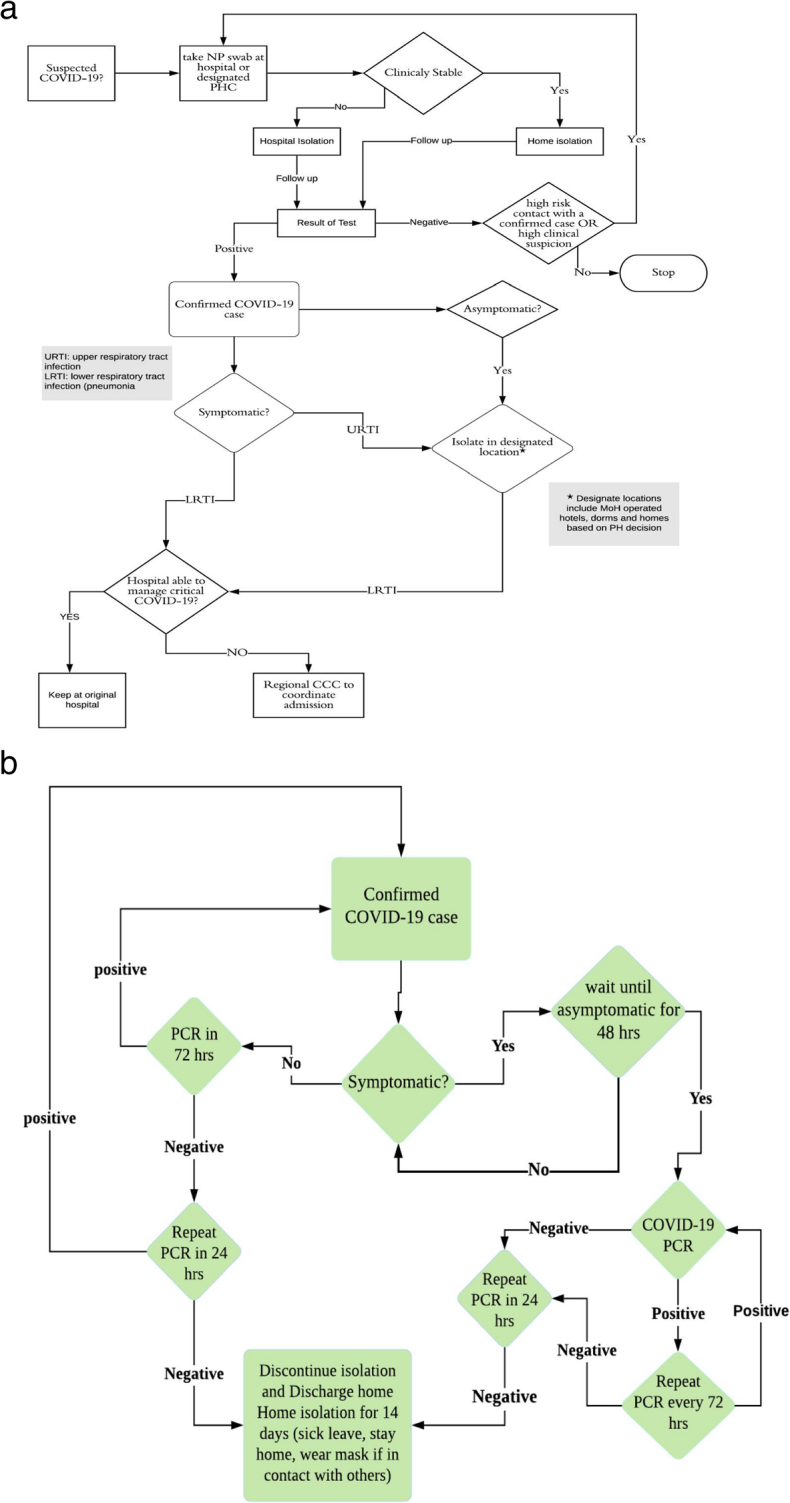
Decision support for the management or discontinuation of isolation for **a.** suspected COVID-19 and **b.** confirmed COVID-19 cases. COVID-19, coronavirus disease 2019

To limit COVID-19 spread, hospitals provided face masks to all employees entering the building, trained the staff on the proper use of personal protective equipment (PPE), and tracked the staff members who became ill and monitored their test results. Additionally, the MOH sponsored a temporary residence for employees working with COVID-19 patients. To allocate the limited bed capacity, hospitals prioritised the services and types of procedures that could be deferred, based on the permissible duration and consequences of the deferral. Alternate plans were created for patients who would be deferred. Updated guidelines were established for triage and the use or denial of ICU services, such as mechanical ventilation. All patients visiting the hospital were screened by visual triage, temperature measurement, and a questionnaire survey (Table 1). Suspected COVID-19 patients were subjected to further examination and laboratory tests (Fig. 1 a). Visitor numbers were restricted to one for each patient. Staff members from abroad who wished to return to the KSA were required to undertake a 14-day home quarantine. Health care providers (HCPs) who delivered care for COVID-19 patients carried on with usual planning and checked themselves for fever and respiratory symptoms. If staff members were not well protected while in close contact, they were placed off duty while an infectiologist evaluated the risk of in-hospital transmission and planned further management. All HCPs underwent fever screening twice a day. Furthermore, resources including reading materials; a helpline; and visual alerts, such as signs and posters at entrances and in strategic places providing instructions on hand hygiene, respiratory hygiene, and cough etiquette were provided [10]. Moreover, medical supplies, such as tissues, waste receptacles, alcohol-based hand sanitisers, and facemasks, were stocked and made available at visual triage. The MOH clearly defined the roles of HCPs (for avoiding the potential chaos on the arrival of a COVID-19 patient) and the institution (for strict infection prevention and control measures for cleaning, laundry, food services, and visits) [10].

**Table 1:** Standard questionnaire for respiratory illnesses

Risk of Acute Respiratory Illnesses	Score	
**A. Exposure Risks**	**Any Patient (Adult or Paediatric)**	
History of foreign travel during the 14 days prior to symptom onset. **OR** Visiting or being a resident of a high-risk area for COVID-19 in the KSA during the 14 days prior to symptom onset ^a^ **OR** Close physical contact with a confirmed case of COVID-19 or MERS-CoV in the past 14 days. **OR** Exposure to camels or camel products (direct or indirect ^b^) in the past 14 days. **OR** Working in a healthcare facility.	**3**	
**B. Clinical Signs and Symptoms and Medical History**	**Paediatric**	**Adult**
1. Fever or recent history of fever	**1**	**2**
2. Cough (new or worsening)	**1**	**2**
3. Shortness of breath (new or worsening)	**1**	**2**
4. Nausea, vomiting, and/or diarrhoea	**–**	**1**
5. Chronic renal failure, CAD/heart failure, immunocompromised status	**–**	**1**
Total score		
**Score ≥ 4: ask the patient to perform hand hygiene and wear a surgical mask, direct the patient through the respiratory pathway, and inform an MD for assessment.**		
**MERS-CoV or COVID-19 testing should only be performed according to the case definition.**		

^a^As determined and announced by the Ministry of Interior or Ministry of Health. Updated regularly on: www.covid19.cdc.gov.sa

^b^Patient or household

*CAD* Coronary artery disease, *COVID-19* Coronavirus disease 2019, *MD* Doctor of medicine, *MERS-CoV* Middle East respiratory syndrome coronavirus

On 6 April, 2020, the government imposed a 24-h quarantine in most cities. Patients were allowed to go to the hospital by ambulance or by calling the emergency medical services. They gave a code for patients and directed them to the nearest hospital. Consequently, most patients reaching the hospital were emergent cases requiring surgical intervention. In the emergency department (ED), all patients were screened using a standard questionnaire (Table 1). Patients who fulfilled the criteria for suspected COVID-19 were isolated, referred to an infectious disease specialist, and tested for the virus, regardless of their main complaints.

### ED for surgical cases

At King Saud University Medical City, Riyadh, visual triage was conducted at the ED entrance for identifying stable COVID-19 at the time of check-in [3]. Patients presenting with respiratory symptoms were separated from other patients by at least 2 m and asked to wear a face mask; sitters were not allowed to accompany patients [11, 12]. Patients who required aerosol-generating procedures were placed in a negative pressure isolation room with high-efficiency particulate air (HEPA) filtration [3, 12]. Movement to and from the patient’s room was limited to HCPs involved in patient care [12].

**Fig. 2:**
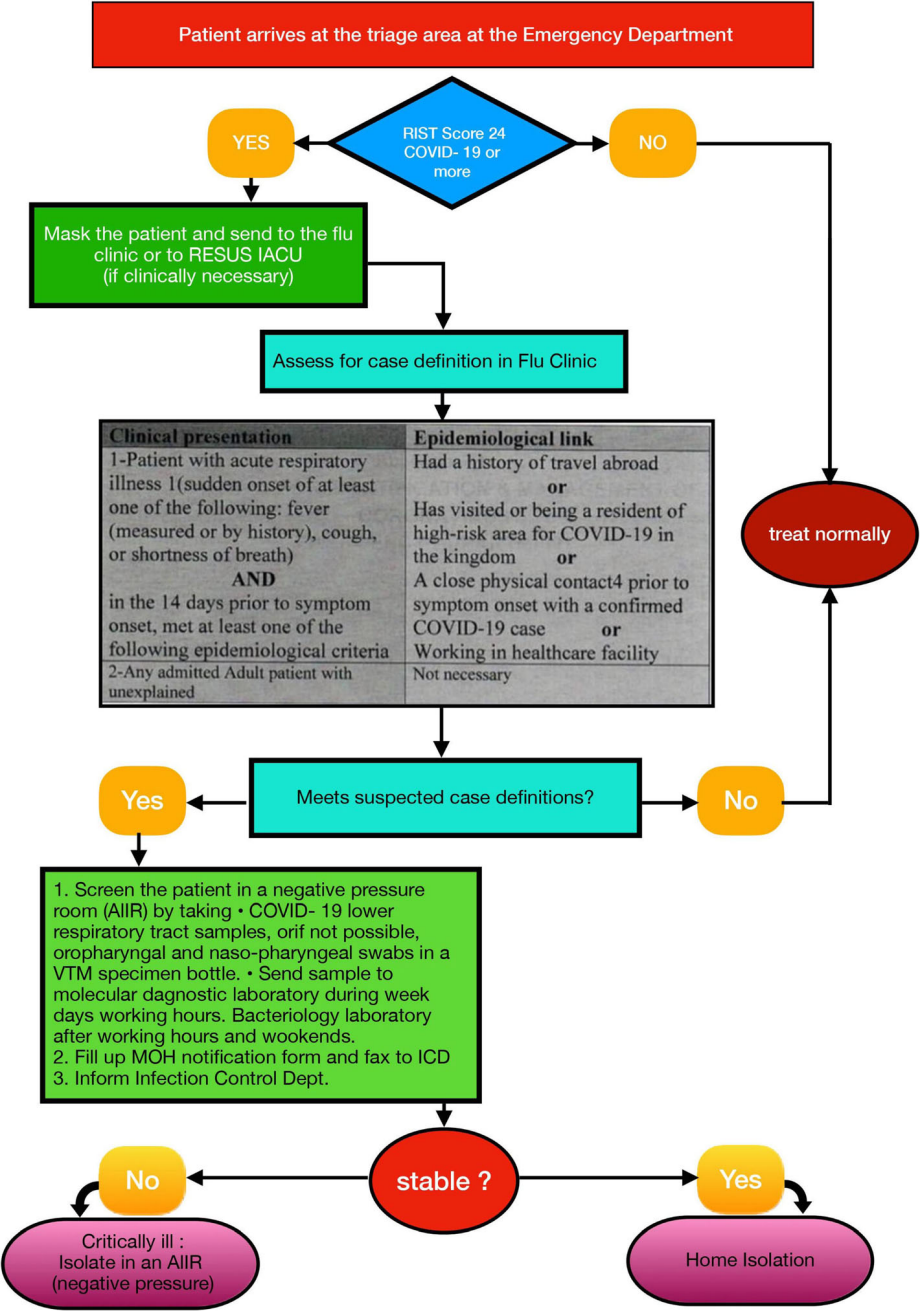
Pathway for the identification and management of COVID-19 cases in the ED ED, emergency department; COVID-19, coronavirus disease 2019.

Stakeholders at the ED developed a protocol for helping the identification and management of patients with confirmed/suspected COVID-19 (Figs. 1 and 2).

### ICU preparation

#### Intensive care contingency planning

The initial action at King Faisal Specialist Hospital and Research Center, Riyadh, was to form a group for handling all critical care issues. The chief officer of this group was a consultant intensivist who reported directly to the command centre chief. The group was composed of highly qualified intensivists with different backgrounds and experiences, including the head of respiratory care, head of critical care nursing, and one critical care clinical pharmacist. We conducted several online meetings to plan our actions and assign roles and responsibilities. The actions are summarised in the following sections:

#### Location and patient flow

Critically ill patients who tested positive for COVID-19 or were highly suspected of having COVID-19 with pending results underwent immediate consultation with the Critical Care COVID-19 team. The team started clinical and bedside management and facilitated transfer to the Critical Care COVID ICU once the results were confirmed.

#### ICU beds: capacity and hardware

We allocated a certain unit in the same COVID-19 building. It is preferable to have as many negative pressure rooms as possible. Negative pressure rooms were equipped with two HEPA filters placed on each side of the bed. Negative pressure rooms could be reserved for aerosol-generating procedures, such as intubation.

To minimise unnecessary movement into patient rooms, rooms were equipped with advanced monitoring systems and security cameras. These monitors allowed the HCPs to control cardiac monitors and ventilators and inspect the patients from outside.

As the number of patients increased, some of the other units were used as COVID-19 ICUs, including medical and surgical ICUs, ORs, post-anaesthesia care units, and cardiac care units. Some initially considered rooms were upgraded by equipping them with cardiac monitors, central monitors at the nursing station, end-tidal CO_2_ monitors, transport monitors, portable suction, ICU beds, point-of-care blood glucose monitors, and vein viewers. Some equipment required for bedside procedures was also obtained, including chest tubes, tracheostomy, cut down, and external ventricular drain insertion trays.

Respiratory care equipment was also ordered, including ICU ventilators (portable and magnetic resonance imaging compatible), video laryngoscopy equipment, end-tidal CO_2_ devices, difficult airway carts, high-flow oxygen therapy equipment, chest physiotherapy precursors, bronchoscopes, nitric oxide tanks, oxygen tanks, and Heliox tanks.

#### Manpower surge planning

To provide adequate manpower, the surge planning was divided into several stages depending on the number of patients. In the first stage, patients numbered between 7 and 12, and we allocated a low-risk consultant intensivist (< 50 years old) to take care of the COVID-19 patients. At this stage, there would be three teams, each with one consultant and one assistant responsible for the care of six patients. In the second stage (around 30 patients), the numbers of consultants and assistant consultants would increase to 10 each. They would form five teams working 12-h shifts. Paediatric intensivists would be available upon request. In the third stage (around 100 patients), we would ask for assistance from our colleagues from the cardiac ICUs and anaesthesia unit to work in critical care areas. They would form 13 teams working 12-h shifts.

During the last stage, when the maximum capacity is reached (around 140 patients), a non-intensivist would be asked to help in critical care areas after taking a crash course in the fundamentals of critical care, with backup help from an intensivist. Overall, 18 teams would be formed. Each team would have one consultant and one assistant consultant taking care of 7–8 patients on average.

#### Logistics and protocol

The Critical Care COVID-19 team developed the following documents and protocols as guidance for all staff members:

#### F.1 COVID-19 CCM team policies and procedures

This is a general document to provide definitions, roles, and responsibilities to all the critical care physicians, nurses, and respiratory technicians working in critical care areas for COVID-19 patients. It includes descriptions of the work location and operational safety huddle that occurs twice daily during shift change.

#### F.2 COVID-19 patient flow

It describes how to transfer patients from the ED to the COVID-19 ICU. It also describes the logistics related to caring for the patient while waiting for swab tests results.

#### F.3 clinical management protocols

This comprises multiple documents that include systematic management, mechanical ventilation, and Pain, Agitation/Sedation, Delirium, Immobility, and Sleep disruption guidelines. Each document describes in detail how to manage critically ill patients and how to provide guidance and protocol. These documents will be valuable during the surge when non-intensivists will be working in the ICU.

#### F.4 intubation and Extubation protocols

These documents are for clinical management during intubation and extubation and simulation planning for both procedures. They describe roles and responsibilities, team dynamics, and the location of each member during the procedures. They also describe a step-by-step approach on how to conduct these procedures safely. Furthermore, we videographed some intubation and extubation simulation sessions to add visual aids for the documents.

#### F.5 early warning signs and cardiac arrest

This helps in the early stratification and detection of critically ill patients. It helps consultants working on the floor, facilitating early ICU transfer for patients potentially requiring mechanical ventilation. It describes the communication process and the transfer of logistics between the floor and ICU. Cardiac and respiratory arrest events are also considered ‘aerosol-generating procedures,’ and a separate document provides guidance on what to do during these events. It also describes the roles and location of each member, such as who should be inside the room and who is to remain outside the room to provide support, together with airway management principles.

### Measures in the Anaesthesia department

At King Abdulaziz University Hospital, Jeddah, we developed crisis outbreak response measures in collaboration with surgical, nursing, and other health service staff. The objectives of these measures are to facilitate the care of COVID-19 patients requiring surgery and to reduce the risk of perioperative viral transmission to HCPs and other patients.

#### Personal protective equipment

PPE were provided to all HCPs in the OR. HCPs and their organisations should review protocols for the correct donning and doffing of PPE. Given the transmission risk with non-invasive ventilation, early endotracheal intubation in patients with acute respiratory failure is recommended [13]**.**

Disposable OR caps and beard covers should be properly worn to reduce the contamination risk via hands or hair, which may have been exposed to droplets. Disposable, fluid-resistant long-sleeved gowns, goggles, and full-face shields should also be provided [13]. A powered air-purifying respirator provides superior protection and may be warranted for airway procedures in COVID-19 patients, given prior cases of SARS-CoV infection in HCPs using N95 masks [13].

#### Minimising the exposure and spread of COVID-19

I. Limit the number of OR team members during intubation [14]. The most experienced anaesthetist available should perform intubation to avoid multiple airway instrumentation.
II. Enforce frequent hand washing for all anaesthesia staff [15]. Hand hygiene should be meticulously performed according to standard guidelines, specifically after removing gloves; after contact with soiled or contaminated areas; before touching the anaesthesia machine or anaesthesia cart, and after every contact with the patient (e.g. placement of thermometer or nasogastric tube). Alcohol-based hand wash gels should be located near every anaesthesia workstation.
III. Use extreme caution when removing and disposing PPE to minimise the risk of self-contamination.

### Practical considerations in Anaesthetic Management of Suspected/confirmed COVID-19 patients

As transmission is hypothetically possible before the appearance of symptoms and because it is difficult to identify and isolate carriers, enforcing a standard practice during airway management for all patients undergoing general anaesthesia (GA) is recommended to minimise exposure to secretions [13]. For confirmed COVID-19 patients who require urgent surgery, the benefit-risk of performing or postponing the procedure needs to be reviewed before the final decision.

When GA is not obligatory, the patient should continue to wear a surgical mask [16]. However, if GA is mandatory, specific considerations should be followed concerning airway management and anaesthesia stage.

#### Pre-induction

Hand hygiene should be actively enforced for all HCPs. PPE should be accessible to all providers; wearing double gloves is recommended [17]. Minimally, N95 masks should be worn while treating all known/suspected COVID-19 cases and any ‘open airway’ cases [13]. Prophylactic antiemetics are recommended to minimise the risks of vomiting and potential viral spread [15].

#### GA induction and airway manipulation

The intubation equipment is arranged in close proximity to the patient, and a plan for its disposal should be in place, which limits the travel of the contaminated equipment [18]. Before anaesthesia induction, a HEPA filter is applied to the patient end of the breathing circuit and another between the expiratory limb and the anaesthetic machine [17]. Standard monitoring is applied, and preoxygenation is performed via a well-fitting face mask [15]. Intubation is performed by the most experienced anaesthesiologist. Fibreoptic intubation is avoided, unless specifically indicated. A video-laryngoscope is recommended for intubation, because it keeps the incubator farther from the patient’s airway [3]. Preoxygenation for a minimum of 5 min with 100% oxygen, followed by rapid sequence induction, is performed [19]. To avoid manual ventilation of the patient’s lungs, rapid sequence induction is considered, and a skilled team should be available to apply cricoid pressure or perform a modified rapid sequence induction, as clinically indicated. The laryngoscope should be immediately re-sheathed post-intubation (double glove technique) [17]. All used airway equipment should be sealed in a double zip-locked plastic bag and removed only for decontamination and disinfection. The Surviving Sepsis Campaign recommends initiation with small tidal volumes (4–8 cc/kg ideal body weight) in adults with COVID-19 infection and acute respiratory distress syndrome receiving mechanical ventilation [20]. All unnecessary disconnections of breathing circuits should be avoided. If disconnection is needed, the endotracheal tube should be clamped. If extensions are needed, they should be attached before induction [21].

Extubation is a more critical event than intubation due to the higher chances of coughing. Nonessential personnel should leave the room; airborne precautions should be followed during extubation [5]. The patient should be extubated in the OR or transferred to the ICU [16]. Post-procedure, allow time for the infectious contamination to be removed. The time period depends on the number of air exchanges per hour in the specific room [22]. The need to leave and re-enter the OR should be minimised. As communication is difficult while wearing PPE, staff should pay the attention to facilitate communication during procedures [23].

After surgery completion, the anaesthetic breathing circuit and soda-lime canister are discarded to eliminate the negligible risk of circuit contamination [15, 23, 24]. When transferring intubated patients, a HEPA filter should be placed between the self-inflating bag and patient at all times. Non-intubated patients should wear a surgical mask at all times. HCPs are advised not to touch environmental surfaces, like doorknobs and elevator buttons, during transport. These actions should be performed by an accompanying security team [21]. Team debriefing is an essential strategy for detecting system defects and improving team performance.

### Operating room management

Original work plans are necessary for developing isolation operating theatres. The plans include infection prevention measures, use of surgical and anaesthetic instruments, careful movement, and responsibilities of each OR team member.

**Fig. 3:**
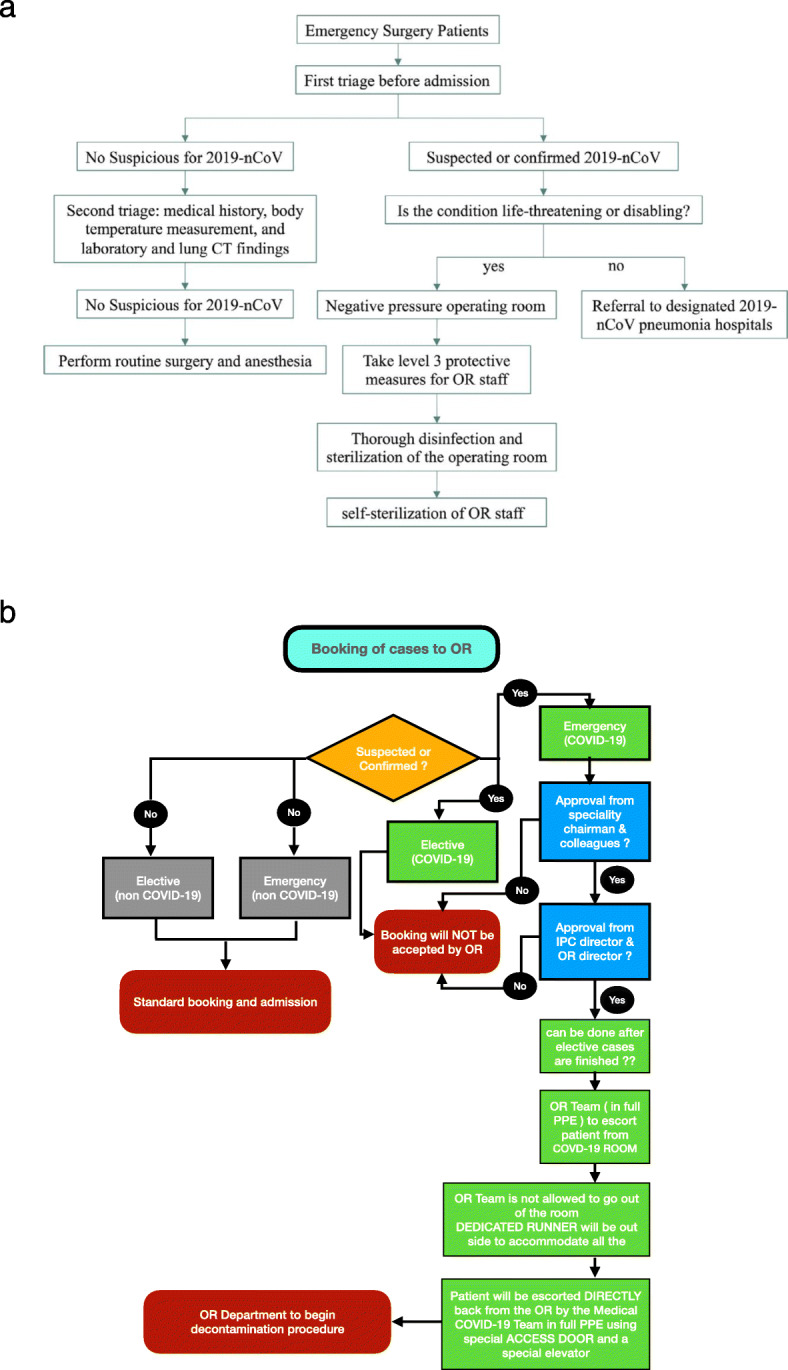
**a** Protocol at the Department of Anaesthesiology at the Wuhan Union Hospital concerning emergency surgical procedures (permission from the copyright holder was taken to reproduce this diagram that has previously been published [25]) **b** Patient flow with a suspected or confirmed COVID-19 patient who is undergoing surgical procedures. COVID-19, coronavirus disease 2019

**Fig. 4:**
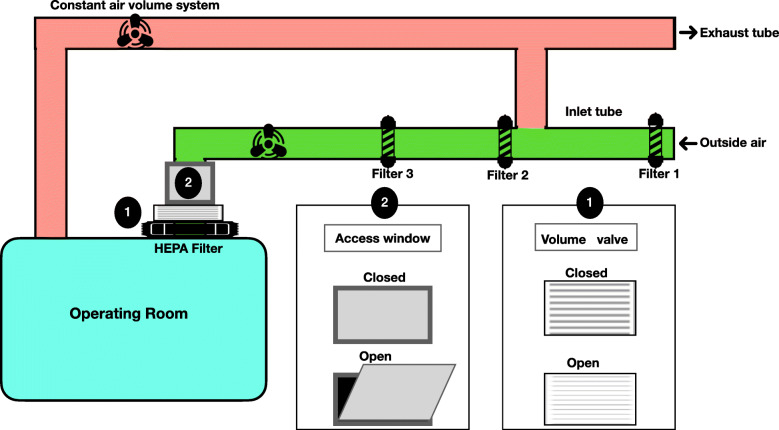
Ventilation system of the operating room

The OR committee at the International Medical Center in Jeddah established guidelines for the management and flow of COVID-19 patients requiring surgery. (Fig. 3.b). A negative pressure OR with the anteroom located on the side of the operation theatre complex, with an exclusive path, is used for all COVID-19 patients. Understanding the airflow within the OR is essential to reduce the infection risk. In each theatre, outside air is allowed in through an inlet tube; air is released outside through an exhaust tube (Fig. 4). Negative pressure is obtained by reducing the amount of air flowing in through the inlet tube and maintaining a steady volume of air outflow through the outlet tube. This can be achieved in two ways: firstly, by closing the blades of the air volume control valve in the inlet tube (Fig. 4). The inflow air volume does not decrease adequately since the valve is not designed to be impenetrable by air. Secondly, an access window is opened in the inlet tube to reduce the inflow volume; this is generally used for tube maintenance (Fig. 4).

COVID-19 cases will be placed at the end of the list. Adequate amounts of medications, fluids, and other materials should be prepared in the OR before initiating the surgical procedure. All built-in machines including portable computers, telephones, and anaesthesia gas machines are protected using a plastic cover. Standard PPE includes surgical scrub suits, surgical gloves, gowns, face shields, and N95 masks. HEPA filters are placed on ventilators and at the patient’s site, connected to the endotracheal tube. Furthermore, the intubation trolley is set in the induction room. Airways should be secured using a video-laryngoscope to avoid repeated airway instrumentation [26].

Each member in the OR has to be assigned a particular function. The security officer is responsible for clearing the corridor, from the admission unit or ICU to the OR, including the elevators. The ward nurses are accountable for transferring the patient to the OR in full PPE. For ICU patients, an allocated transport ventilator is used. The gas machine should be turned off, and the endotracheal tube should be clamped to avoid aerosolisation.

During the surgical procedure, a circulating nurse remains outside the OR wearing PPE to help in handling specimens and additional equipment. Gowns and gloves should be discarded in the anteroom; team members must perform hand hygiene before leaving the anteroom. Furthermore, any powered air-purifying respirator should be moved outside the anteroom. According to the healthcare organisation policy, the OR is cleaned after exposure to confirmed COVID-19 patients. A further precaution involves decontaminating the OR after exposure using a hydrogen peroxide vaporiser.

### Special consideration of different Despines

#### General surgery

Under the present circumstances, hospitals in the KSA decided to handle elective surgical cases. Urgent cases (cancer and transplants) were also postponed. Moreover, polyclinics providing dental and cosmetic services were closed. The Saudi General Surgery Society, in collaboration with the Saudi Patient Safety Center, categorised surgical procedures into four priority-based groups (Supp. 1): Priority 1, a patient needs to be operated on within 24 h (acute appendicitis); Priority 2, a patient needs to be operated on within 1–7 days (acute cholecystitis); Priority 3, a patient needs to be operated on within 7–30 days (gastrointestinal oncology); and Priority 4, patient needs to be operated on within > 30 days (benign cases: anal conditions and hernias). With an increasing number of positive cases, some hospitals have closed their ED and have been assigned to receive only COVID-19 cases.

#### Orthopaedic surgery

In the KSA, the American Academy of Orthopaedic Surgeons guidelines are followed (20). They have deferred elective cases; surgeries are performed for limb-threatening and life-threatening diseases, disease with a painful nature, and diseases that can cause functional disability. Cases not included in the mentioned categories are discussed on a case-by-case level.

#### Neurosurgery

The Saudi Association of Neurological Surgery guidelines [27] recommend delaying all elective surgeries involving the brain and spine, except those involving life-threatening situations and rapidly evolving neurological deficits. Brain infection and cerebrospinal fluid problems, such as hydrocephalous or leaking meningocele or encephalocele, represent serious problems and should be treated immediately. Patients with traumatic brain injuries, such as subdural or epidural hematoma, ruptured cerebral aneurysm, or dural fistulas, would be operated on if they developed disturbed consciousness or neurological deficits. They should be managed either surgically or by embolisation, if feasible. Endoscopic skull base surgery, like the transsphenoidal approach for pituitary surgery, is considered to be a very high-risk procedure. Subsequently, these cases are managed using the transcranial approach. The Saudi Spine Society guidelines [28] recommend stopping elective spinal surgeries. Emergent surgery is reserved for patients with neurological deficits related to radiologically confirmed neural element compression or spinal instability, regardless of the cause. Some cases should be resolved within a reasonable period (cervical myelopathy, intractable pain related to disc herniation, and postoperative infection).

#### Obstetrics and gynaecology

The Obstetrics and Gynaecology Department at the King Saud University Medical City developed a pathway for emergency gynaecology and obstetrics procedures for COVID-19 patients based on the Royal College of Obstetrics and Gynaecology guidelines (Supp. 2) [29]. All non-emergency gynaecological surgeries are delayed, except in cancer and urgent cases wherein the delay can produce adverse effects (fistula cases) (Supp. 3). One of the major objectives of the obstetric taskforce is to efficiently communicate with patients to ensure they do not miss any scheduled appointments and planned elective caesarean section. After curfew implementation, patients were worried about coming to hospitals; some patient presented overdue, where cases were nearly missed. Hospital administration reacted promptly and sent mass messages to all obstetrics patients to enforce attendance at hospitals and to not miss any appointments. To facilitate patient transportation, the government provided electronic services for patients to request transport permits in collaboration with the hospitals, the Ministry of the Interior and the medical team to ensure that patient care was not affected by curfew orders. In the OR, caesarean section under neuraxial anaesthesia rather than under GA was encouraged. Suspected/confirmed COVID-19 patients and those done under the effect of GA were all performed in a negative pressure room [30, 31].

Contrastingly, gynaecological cases were easier to manage. Initially, cases involving cancer and some premalignant conditions were excluded from this rule, because the risk of progression to malignant disease was considered high; some patients were already on the waiting list. When the hospital announced its first positive case, it also decided to delay cancer cases. According to the infection control policy, this was considered a phase three situation, wherein only critical surgeries were allowed, such as those for trauma. Given that most gynaecological procedures (cancer and emergency cases) are minimally invasive, this was a debatable topic. Although hospital policies were followed, and after ensuring cases are urgent and no other equivalent therapeutic alternative was available, these cases were all performed in negative pressure rooms and laparotomy cases only were permitted in positive pressure rooms despite the fact that these laparoscopic procedures could be performed in regular rooms with suitable PPE precautions with the minimum OR time possible [32]. The King Saud University Medical City viewed all admitted patients as positive for COVID-19; this measure kept the obstetrics and gynaecology service COVID-free.

### Continued education and awareness of HCPs and simulation

All HCPs received training in infection control and prevention strategies and in procedures for donning and doffing PPE [19]. Employees dealing with COVID-19 patients compulsorily 1) received comprehensive training on proper PPE use—i.e., how to don and doff PPE and its limitations, maintenance, and disposal; and 2) demonstrated competency in performing appropriate infection control practices and procedures [33].

HCPs are mandated to undergo respirator fit testing using N95 masks. They attended a simulation on how to properly don and doff powered air-purifying and N95 respirators. At our institution we adopted the ‘aerosol-box’ and ‘transparent drape’ methods as the safest ways for intubating patients with COVID-19 to prevent cross-contamination and infection of HCPs. More than one donning and doffing method is acceptable; we followed the steps illustrated in (Table 2) [33].

To achieve maximum safety, simulation sessions were conducted to train all staff members on how to intubate patients using both the ‘aerosol-box’ and ‘transparent drape’ methods. For extubation, we adopted the ‘Heliox bag’ as the safest way for extubation to protect HCPs. All staff members including respiratory technicians and registered nurses are required to attend training on how to use the ‘Heliox bag’ for extubation.

**Fig. 5:**
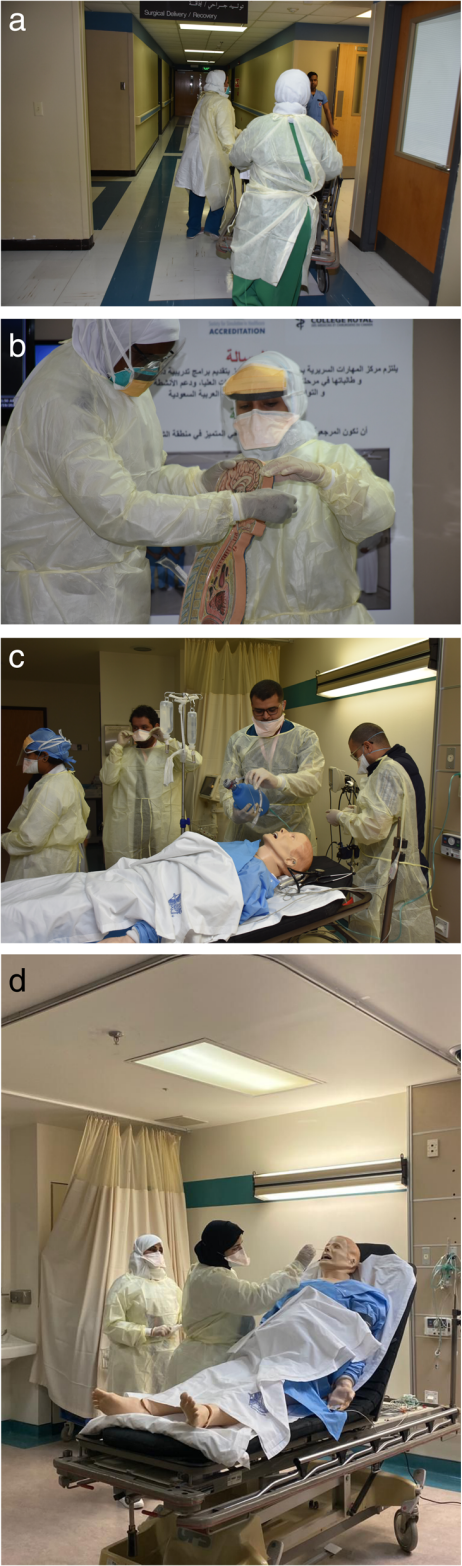
Using medical simulation for COVID-19 preparation at the King Abdulaziz University Hospital clinical skills centre (**a**) transport of a patient with suspected COVID-19; (**b)** donning and doffing personal protective equipment; **c.** intubation simulation scenario; (**d**) simulation for nasopharyngeal swab collection. COVID-19, coronavirus disease 2019

The King Abdulaziz University Hospital Clinical Skill Centre developed multiple simulation scenarios and sessions for performing proper intubation, handling PPE, and dealing with COVID-19 patients (Fig. 5).

At the King Saud University Medical City simulation centre, multiple simulation drills were performed in ORs to verify the readiness of the flow and structure for receiving COVID-19 patients. Furthermore, several drills for suspected cases were performed for trainees and nurses who attended and practiced using PPE effectively.

As an effort to train non-intensivists, members of the ICU department participated in writing online lectures on the fundamentals of critical care. The King Faisal Specialist Hospital and Research Center in Riyadh developed a series of lectures focused on airway management, respiratory failure, cardiovascular failure and shock, sedation management, and acute kidney injury. The lectures were available online for those who wished to participate during the surge. Each participant would receive a certificate upon completion of these lectures.

## Discussion

In reaction to the COVID-19 pandemic, multiple measures have been taken to decrease hospital-acquired infections and allocate limited hospital resources, especially ICU beds and ventilators. In this review, we present the protocols and share our experience of contingency planning in tertiary hospitals in the KSA during this crisis. We followed the WHO 2007 guidelines regarding infection prevention and control of epidemics and pandemics in healthcare facilities by providing appropriate PPE to HCPs and prioritising suitable medical services and procedures [34]. Other administrative measures in the hospital environment included the screening of patients and HCPs entering the hospital, providing a residence sponsored by the MOH for employees working with COVID-19 cases, and enforcing a 14-day home quarantine for all employees returning from abroad. To our knowledge, no such planning review, including guidelines and protocols, has been published.

Surgical emergencies have been described during previous coronavirus epidemics; however, the roles and responsibilities of the emergency medical system have received little attention [35]. The clinical recommendations for emergency practice to reduce infection transmission [35] are as follows: to provide a reserved area with diagnostic materials for all suspected cases; to provide early results of diagnostic tests to avoid the crowding of patients requiring care for other illnesses; to provide isolation rooms with negative air pressure and identify any healthcare deficit and favour physical barriers between patients and HCPs, with a distance of approximately 2 m; and to provide an additional backup supply of protective materials and PPE [36].

The Department of Anesthesiology at Wuhan Union Hospital drafted a protocol to manage emergency surgical procedures for COVID-19 patients [25] (Fig. 3.a). All anaesthesiology societies with experience with SARS-CoV-2 have published similar peri-operative measures for the management of COVID-19 cases [37–39]. To protect HCPs, Chen et al. [37] prioritised the use of GA in surgeries in those with confirmed SARS-CoV-2 to reduce airborne droplets. Lie et al. [40] considered local anaesthesia to preserve respiratory function and avoid possible aerosolisation. Further studies are necessary to assess the implications of different types of anaesthesia in this population. As stated previously, our experience during this pandemic was different in terms of connecting two ORs: the first was used as a theatre and the second was used as the anteroom, where the circulating nurse stored and collected surgical equipment in accordance with the guidelines of the US Centers for Disease Control and Prevention [41]. Ti et al. [26] explained intra- and inter-hospital patient transfer and the responsibilities of each OR team member; their suggestions support our protocol regarding suspected COVID-19 cases. To date, several studies have highlighted safety measures in the OR and stratification of surgical interventions. Firstenberg et al. [42] presented their experience with a 77-year-old patient diagnosed with COVID-19 who required emergency cardiac surgery for acute aortic syndrome. To minimise the risk of infection, they provided a protocol regarding transporting the patient from the ICU to the OR, preoperative preparations, and OR cleaning procedures. Likewise, Stahel showed that elective procedures may contribute to COVID-19 transmission [43]. A decision-making algorithm for risk-stratification of elective procedures based on the indication of surgical care and predicted resource consumption during the current COVID-19 pandemic was suggested [43].

The Royal College of Surgeons of Edinburgh issued the following guidelines for the surgical response to COVID-19: consider every patient as potentially COVID-19 positive; when performing an intestinal procedure, stoma formation should be chosen over anastomosis to decrease the need of postoperative intensive care; it is very important to weigh the risks and benefits of laparoscopy in selected patients, considering potential viral transmission to the OR staff; and whenever possible, non-surgical management should be considered [44].

The American College of Surgeons guidelines recommend conservative management for most emergency surgical situations. This should be considered according to the patient’s condition and surgeon’s decision. For patients with uncomplicated acute appendicitis, a conservative approach can be considered. Short-stay laparoscopic appendectomy can help decrease postoperative hospital stay [45]. For complicated appendicitis and appendicular abscess, the original practice is implemented [45]. Ascending cholangitis patients can respond to adequate resuscitation and broad-spectrum intravenous antibiotics [45]. If this approach fails to improve the patient’s condition or if the patient has septic shock, endoscopic retrograde cholangiopancreatography with sphincterotomy is indicated. Patients with acute cholecystitis should be assessed for surgery risk. If the patient is healthy and fit, laparoscopic cholecystectomy can be performed to reduce the length of hospital stay [45]. If the patient is at high-risk for the procedure or in septic shock, percutaneous cholecystostomy tube placement along with intravenous antibiotic administration should be performed [45]. It is important to weigh the risk of postponing surgeries in gastrointestinal oncology patients, who may need postoperative ICU support. Surgeries for all other benign conditions can be delayed for months without a life-threatening risk [45]. The practice in KSA is parallel to these practices [44, 45].

Regarding orthopaedic surgery, we follow the guidelines published by the University of Pennsylvania and recommended by the American Academy of Orthopedic Surgeons [46]. Concerning brain pathologies, the Congress of Neurosurgical Surgeons published recommendations and a treatment algorithm, which is a useful tool that helps neurosurgeons synthesise neurosurgical recommendations and practice [47]. On 31 March, 2020, JAMA Otolaryngology–Head & Neck Surgery published its recommendation regarding the necessary precautions during skull base surgery [48]. On 3 April, 2020, the North American Spine Society published its recommendations and guidelines for the management of spine pathologies during the pandemic [49].

Obstetric services are especially difficult to suspend, delay, or control. Delaying these procedures will not only put maternal and foetal lives at risk but will also convert these elective cases to emergency cases, involving high morbidity and mortality rates [29, 30, 50].

Many published studies have described the potential of simulation to improve hospital flow, HCP knowledge and skills, and patient safety. Dieckmann et al. stated that simulation has helped in managing the global health crisis in 2020 and will similarly help in battling future pandemics [51].

## Conclusions

The recommendations described in this paper are based on international clinical practice and have been modified according to the healthcare system in the KSA. Changes arising at the healthcare system level in relation to the COVID-19 pandemic affect all hospital services. Changes in the OR setting involve multiple levels and many specialties, making the coordination and adaptation of new recommendations challenging. We recommend each hospital to develop a national guideline based on the healthcare system level and infrastructure facilities, considering the international guidelines. Furthermore, we recommend that the government should assign a hospital in each city mainly for urgent, non-infection, cases, such as cancer cases. Delaying treatment in these cases will result in unacceptable morbidity and mortality and providing treatment in the same COVID-19 centre will greatly increase the risk of transmission.

## Supplementary information

**Additional file 1.**

## Data Availability

Not applicable.

## Abbreviations

COVID-19: Coronavirus Disease 2019
ED: Emergency Department
GA: General Anaesthesia
HCP: Health Care Provider
HEPA: High-Efficiency Particulate Air
ICU: Intensive Care Unit
KSA: Kingdom of Saudi Arabia
MOH: Ministry of Health
OR: Operating Room
PPE: Personal Protective Equipment
SARS-CoV-2: Severe Acute Respiratory Syndrome Coronavirus 2
WHO: World Health Organization
