# Supplementary material for: Safety measures for COVID-19: a review of surgical preparedness at four major medical centres in Saudi Arabia
Source: Patient Saf Surg. 2020 Sep 5;14:34. doi: 10.1186/s13037-020-00259-1 (PMC7474573; doi:10.1186/s13037-020-00259-1)
Supplement: Supplementary file 1 — Additional file 1. [file 13037_2020_259_MOESM1_ESM.docx]

**Safety measures for COVID-19: a review of surgical preparedness measures at four major medical centres in Saudi Arabia.**

Mohammad A. Alsofyani^1^, Ahmed Bashawyah^2^, Mohammed Bawazeer^3^, Khalid Akkour^4^, Sultan Alsalmi^5^, Abdu Alkhairy^6^, Nayef Bin Dajim^7^, Salahaddeen Khalifah^7^, Ibrahim A. Almalki^8^, Farid Kassab^9^, Mohammad Barnawi^10^, Mosfer Almalki^11^, Mohammed Alharthi^12^, Majed Alharthi^13^, Abdulaziz Almalki^14^, Abdullah H. Almalki^15^ Anouar Bourghli^16^, Ibrahim Obeid^17^, Haifaa M. Malaekah^18^

1. Orthopedic Department, College Of Medicine And University Hospital, University Of Hail, P.O. Box 2440, Hail, Kingdom Of Saudi Arabia.
2. Anesthesiology Department, College Of Medicine And King Abdulaziz University Hospital, Jeddah, Kingdom Of Saudi Arabia.
3. Critical Care Medicine, King Faisal Specialist Hospital And Research Center, Riyadh, Kingdom Of Saudi Arabia.
4. Obstetrics And Gynecology Department, King Saud University, Riyadh, Kingdom Of Saudi Arabia.
5. Department Of Neurosurgery, Imam Abdulrahman Bin Faisal University, Dammam City, Kingdom Of Saudi Arabia.
6. Department Of Neurosurgery, King Faisal Medical City, Abha, Kingdom of Saudi Arabia.
7. Neuroscience Center, King Abdullah Medical City, Makkah, Kingdom Of Saudi Arabia.
8. Infection Control Department, Mental Health Hospital, Taif, Kingdom Of Saudi Arabia.
9. Musculoskeletal Center Of Excellence, International Medical Center, Jeddah, Kingdom Of Saudi Arabia.
10. Anesthesia Department, College Of Medicine And University Hospital, Albaha University, Albaha, Kingdom Of Saudi Arabia.
11. Hematology Oncology Department, King Faisal Specialist Hospital And Research Center, Jeddah, Kingdom Of Saudi Arabia.
12. Biochemistry And Molecular Medicine Department, College Of Medicine, Taibah University, Madinah, Kingdom Of Saudi Arabia.
13. General Surgery Department, Security Forces Hospital, Makkah, Kingdom Of Saudi Arabia.
14. Urology Department, King Faisal Hospital, Taif, Kingdom Of Saudi Arabia.
15. Physical Medicine And Rehabilitation, Armed Forces Rehabilitation Centers, Taif, Kingdom Of Saudi Arabia.
16. Orthopaedic And Spinal Surgery Department, Kingdom Hospital, Riyadh, Kingdom Of Saudi Arabia.
17. Spine Surgery Department, Specialist Terrefort Clinic, 33520 Bruges, France.
18. General Surgery Department, King Abdullah Bin Abdulaziz University Hospital, Princess Nourah Bint Abdulrahman University, Riyadh, Kingdom Of Saudi Arabia.

**Correspondence to:** Haifaa M. Malaekah

General Surgery Department, King Abdullah Bin Abdulaziz University Hospital, Princess Nourah Bint Abdulrahman University, Riyadh, Kingdom Of Saudi Arabia.

Phone: 00966598979986

Email: [h_malaika@hotmail.com](mailto:mohd.alsofyani@gmail.com)

**Supplementary 1**


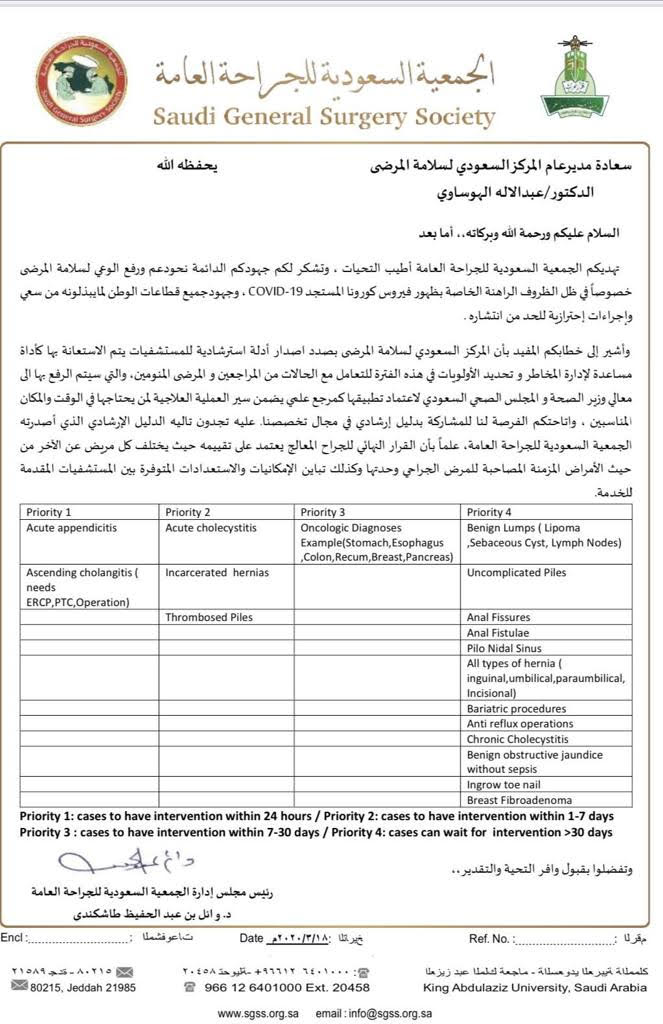


**Supplementary 2**

**Supplementary 3**: FACS guidelines as suggested from Temple University for handling the scheduling of OB/Gyn surgical cases during COVID19 pandemic which was followed at the King Saud University Medical City

| **Emergency surgeries (no delay)**   - Ectopic pregnancy - Spontaneous abortion - Adnexal torsion - Rupture tubal-ovarian abscess - Tubal-ovarian abscess not responding to conservative therapy - Acute and severe vaginal bleeding - Cesarean section - Emergency cerclage of the cervix based on pelvic exam/ultrasound findings |
| --- |
| **Surgeries that if significantly delayed could cause significant harm.**   - Cancer or Suspected cancer   - Ovarian, tubal or peritoneal cancer   - Ovarian masses cancer is suspected   - Endometrial cancer and endometrial intraepithelial neoplasia   - Cervix cancer   - Vulvar cancer   - Vaginal cancer   - Gestational trophoblastic neoplasia - Cerclage of the cervix to prevent premature delivery based on history - Pregnancy termination (for medical indication or patient request) |
| **Surgeries that could be delayed for a few weeks**   - Chorionic villus sampling/amniocentesis (CVS is performed between 11 and 14 weeks of gestation; amniocentesis is performed 15-22 weeks of gestation) - D&C with or without hysteroscopy for abnormal uterine bleeding (pre- or postmenopausal) when cancer is suspected - Cervical conisation or Loop Electro-Excision Procedure to exclude cancer - Excision of precancerous or possible cancerous lesions of the vulva |
| **Surgeries that can be delayed several months**   - Sterilisation procedures (e.g., salpingectomy) - Surgery for fibroids (sarcoma is not suspected)   - Myomectomy   - Hysterectomy - Surgery for endometriosis, pelvic pain - Surgery for adnexal masses that are most likely benign (e.g., dermoid cyst) - Surgery for pelvic floor prolapse - Surgery for urinary and/or faecal incontinence - Therapeutic D&C with or without hysteroscopy with or without endometrial ablation for abnormal uterine bleeding and cancer is not suspected - Cervical conisation or Loop Electro-Excision Procedure for high grade squamous intraepithelial lesions - Infertility procedures (e.g., hysterosalpingograms, most elective embryo transfers) - Genital plastic surgery - Excision of condyloma acuminata (if cancer is not suspected) |

D&C: Dilation and curettage

**Supplementary 4 .** Method of donning and doffing PPE gear.

| **How to Put On (Don) PPE Gear** | **How to Take Off (Doff) PPE Gear** |
| --- | --- |
| 1. **Identify and gather the proper PPE to don.** Ensure choice of gown size is correct. 2. **Perform hand hygiene using hand sanitizer.** 3. P**ut on isolation gown.** Tie all of the ties on the gown. Assistance may be needed by other HCP. 4. **Put on NIOSH-approved N95 filtering face piece respirator or higher (use a facemask if a respirator is not available).** If the respirator has a nosepiece, it should be fitted to the nose with both hands, not bent or tented. Do not pinch the nosepiece with one hand. Respirator/facemask should be extended under chin. Both your mouth and nose should be protected. Do not wear respirator/facemask under your chin or store in scrubs pocket between patients.    1. **Respirator:** Respirator straps should be placed on crown of head (top strap) and base of neck (bottom strap). Perform a user seal check each time you put on the respirator.    2. **Facemask:** Mask ties should be secured on crown of head (top tie) and base of neck (bottom tie). If mask has loops, hook them appropriately around your ears. 5. **Put on face shield or goggles.** Face shields provide full face coverage. Goggles also provide excellent protection for eyes, but fogging is common. 6. **Perform hand hygiene before putting on gloves.** Gloves should cover the cuff (wrist) of gown. 7. **HCP may now enter patient room.** | 1. **Remove gloves.** Ensure glove removal does not cause additional contamination of hands. Gloves can be removed using more than one technique (e.g., glove-in-glove or bird beak). 2. **Remove gown.** Untie all ties (or unsnap all buttons). Some gown ties can be broken rather than untied. Do so in gentle manner, avoiding a forceful movement. Reach up to the shoulders and carefully pull gown down and away from the body. Rolling the gown down is an acceptable approach. Dispose in trash receptacle. 3. **HCP may now exit patient room.** 4. **Perform hand hygiene.** 5. **Remove face shield or goggles.** Carefully remove face shield or goggles by grabbing the strap and pulling upwards and away from head. Do not touch the front of face shield or goggles. 6. **Remove and discard respirator (or facemask if used instead of respirator).** Do not touch the front of the respirator or facemask.    1. **Respirator:** Remove the bottom strap by touching only the strap and bring it carefully over the head. Grasp the top strap and bring it carefully over the head, and then pull the respirator away from the face without touching the front of the respirator.    2. **Facemask:** Carefully untie (or unhook from the ears) and pull away from face without touching the front. 7. **Perform hand hygiene after removing the respirator/facemask and before putting it on again if you workplace is practicing reuse.** |

HCP: health care providers
